# Money and Trusteeship

**Published:** 1917-02-17

**Authors:** 


### MONEY AND TRUSTEESHIP.

How many people are there, whether they are wealthy or poor, who realise that every human being enters this world as a trustee, for his own life, for the blessings of the health with which he is born, for the brains and talents he may possess, and for the soul, which as years grow needs continuous cultivation and opportunities for expression by personal service? How many Londoners are there to-day, who know that, during the thirty-two years from 1885 to 1917, a revolution took place in the Metropolis, touching generosity in gifts and the provision for the sick? We have not space to-day to enforce, by historical detail, the actual happenings and results which prove the thesis just enunciated. The record of this must follow later. To-day we have the privilege to deal with the life of a great citizen of London, great in the sense of his actual work, almost entirely apart from the apprehension of his fellow-citizens, who died, last week, full of years and continuous personal service, aged ninety-one.

Mr. Peter Reid, the father of the Stock Exchange, was born in Perth, where he was educated, a fact which adds one more confirmation to many, of the much greater efficiency of the educational methods in Scotland, compared with England. He came to London as a youth at the time of the great railway boom, and found his first employment as clerk in the office of a railway promoter. He showed remarkable ability, and immediately after the boom, young as he was, was called as a witness at a special sitting in private of a Committee presided over by the Lord Chancellor of the day. Through this and his railway work he became acquainted with Philip Cazenove, a memorable member of the Stock Exchange, entered his office, rose to be a partner and attained to a position of complete confidence with the principal financiers and capitalists of his day. He was an active member of the Stock Exchange for thirty-one years, in the course of which he showed his astuteness, at the time of the Overend and Gurney crash, by refusing the request of a powerful client, because it was not business. This saved his firm from grave loss. Mr. Peter Reid earned and deserved his fortune, and in 1884, when he considered he had enough, he retired from business, bought himself an annuity, and devoted the bulk of his fortune to the building up of important charities, and the supplementing of the work of several of the greater London hospitals, by founding, erecting, organising and endowing the Hospital Convalescent Home at Parkwood, Swanley, Kent. For thirty-three years after his retirement, Mr. Peter Reid actively pursued the practical purpose to which he had devoted his money, the use of which he controlled and supplemented with wisdom, based on a life's experience.

Mr. Peter Reid had a charming personality. He was one of the most attractive, genial, companionable, cheerful, able and unselfish men of his time. He appreciated fully and gratefully that he was a trustee for his own life, and that all his possessions were given to him in trust. He has set a noble example of the fruitfulness and value of a clear apprehension of each man's responsibility to his Creator of the debt which every man owes, as the possessor of the power to make money fruitful in its yield of succour, the restoration of health, and the power to earn and to live, for the humblest and most defenceless of the race. He was a very dear friend to his friends. The memorial service last Tuesday was attended by his contemporaries who have travelled a long way on life's journey. The simplicity of the service, the beauty of the music, the sincere, whole-hearted conduct of both by the pastor and the organist, and the spirit of reverence and worship which pervaded the atmosphere, will be long remembered. It is a worthy, exemplary, and notable fact, that, Mr. Peter Reid has been buried at Parkwood, where he had spent so many fruitful days in personal service, and where his memory will be treasured so long as his institution endures.

